# Supplementary material for: Aqueous phase conversion of CO2 into acetic acid over thermally transformed MIL-88B catalyst
Source: Nat Commun. 2023 May 17;14:2821. doi: 10.1038/s41467-023-38506-5 (PMC10192334; doi:10.1038/s41467-023-38506-5)
Supplement: Supplementary file 1 — Supplementary Information [file 41467_2023_38506_MOESM1_ESM.pdf]

Supplementary Information

Aqueous phase conversion of CO_2 into acetic acid over thermally transformed MIL-88B catalyst

Waqar Ahmad[1], Paramita Koley[1#], Swarit Dwivedi[1,2], Rajan Lakshman[1], Yun Kyung Shin[2], Adri C. T. van Duin[2], Abhijit Shrotri[3], Akshat Tanksale*[1]

[1] *Department of Chemical and Biological Engineering, Monash University, Clayton 3800, Australia*

[2] *Department of Mechanical Engineering, The Pennsylvania State University, University Park, PA, USA*

[3] *Institute for Catalysis, Hokkaido University, Sapporo 001-0021, Japan*

*Corresponding author: akshat.tanksale@monash.edu

Characterization Results

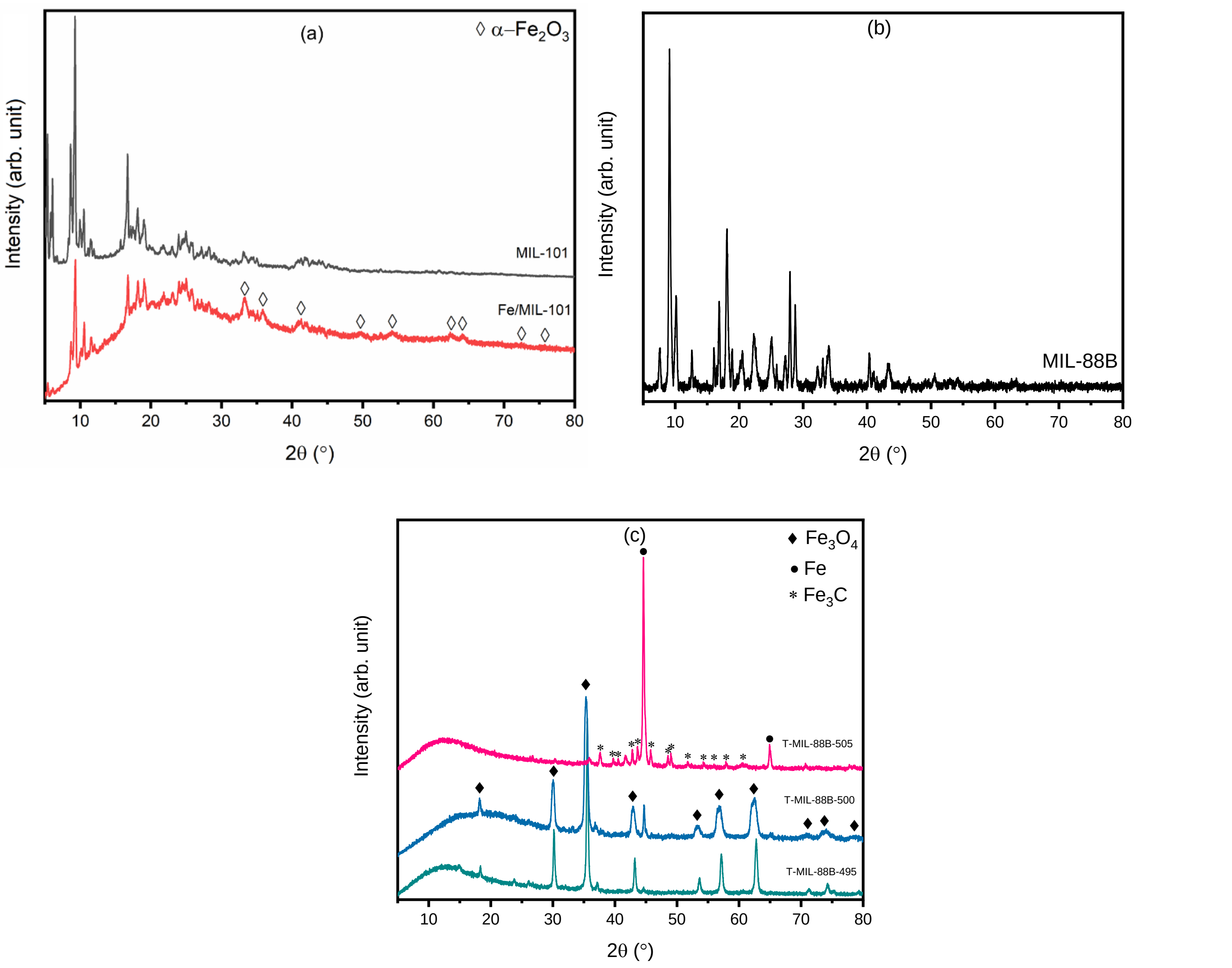

Fig. S1. PXRD pattern of synthesized (a) MIL-101 and Fe/MIL-101 catalyst, (b) MIL-88B, and (c) T-MIL-88B treated at different temperatures

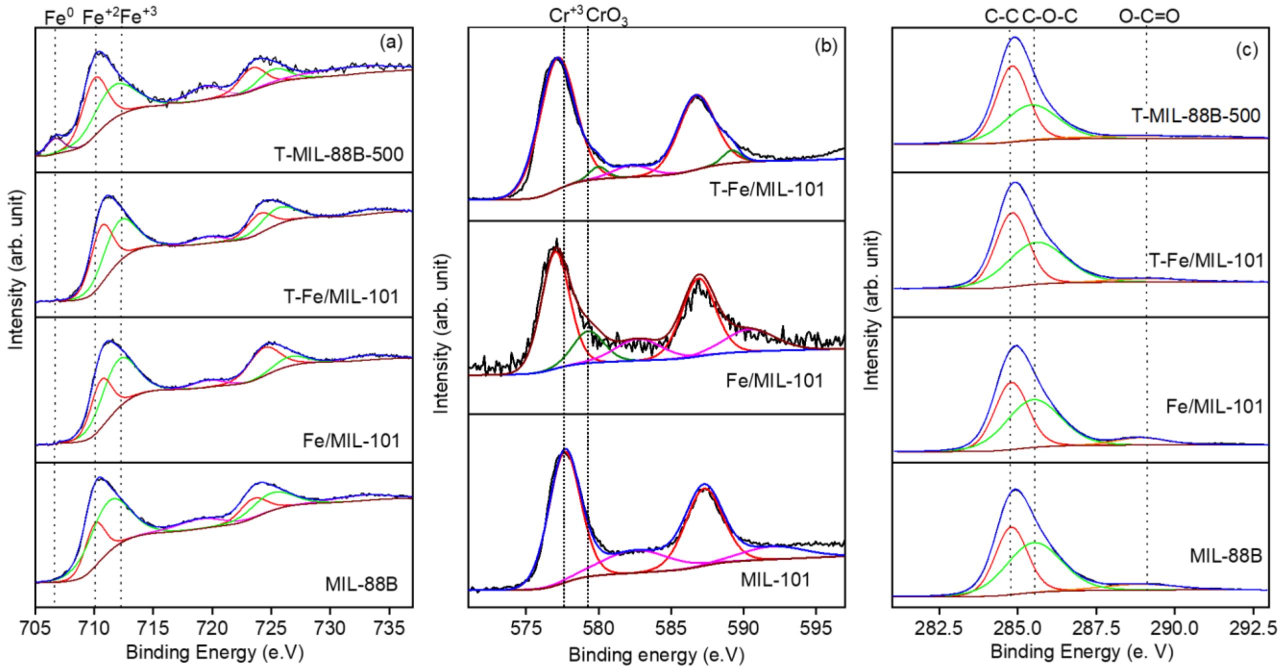

Fig. S2: Narrow scan XPS spectra of (a) Iron 2p, (b) Chromium 2p, and (c) Carbon 1s for the studied catalysts.

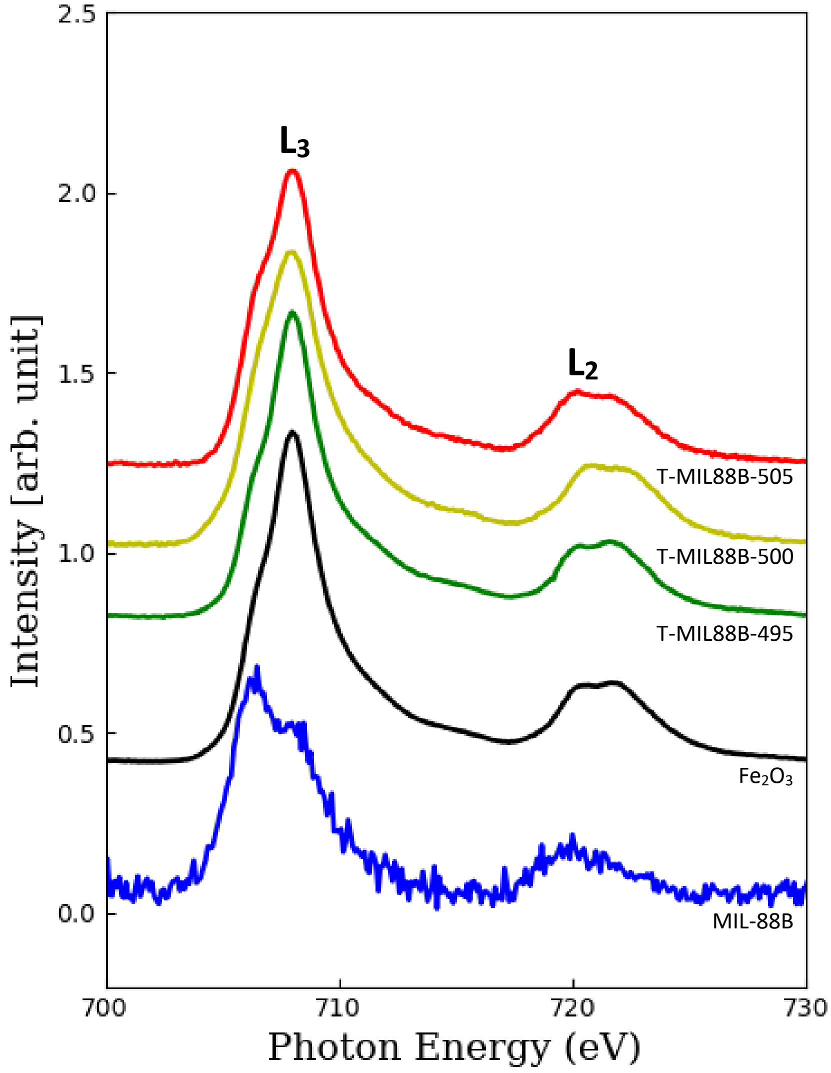

Fig. S3: $L_{2,3}$-edge Fe X-ray absorption near edge spectroscopy (XANES) of MIL-88B and thermally transformed MIL-88B at different temperatures

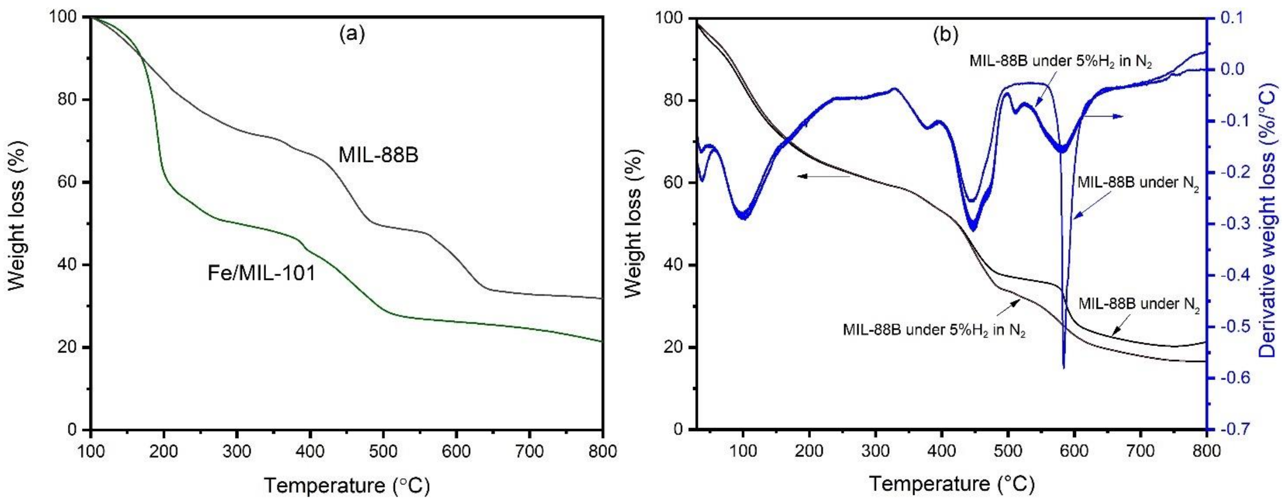

Fig. S4: (a) Thermogravimetric (TG) analysis of Fe/MIL-101 and MIL-88B under Ar atmosphere, and (b) TG and derivative thermogravimetric (DTG) analysis of MIL-88B under 5%H_2/N_2 and pure N_2 atmosphere.

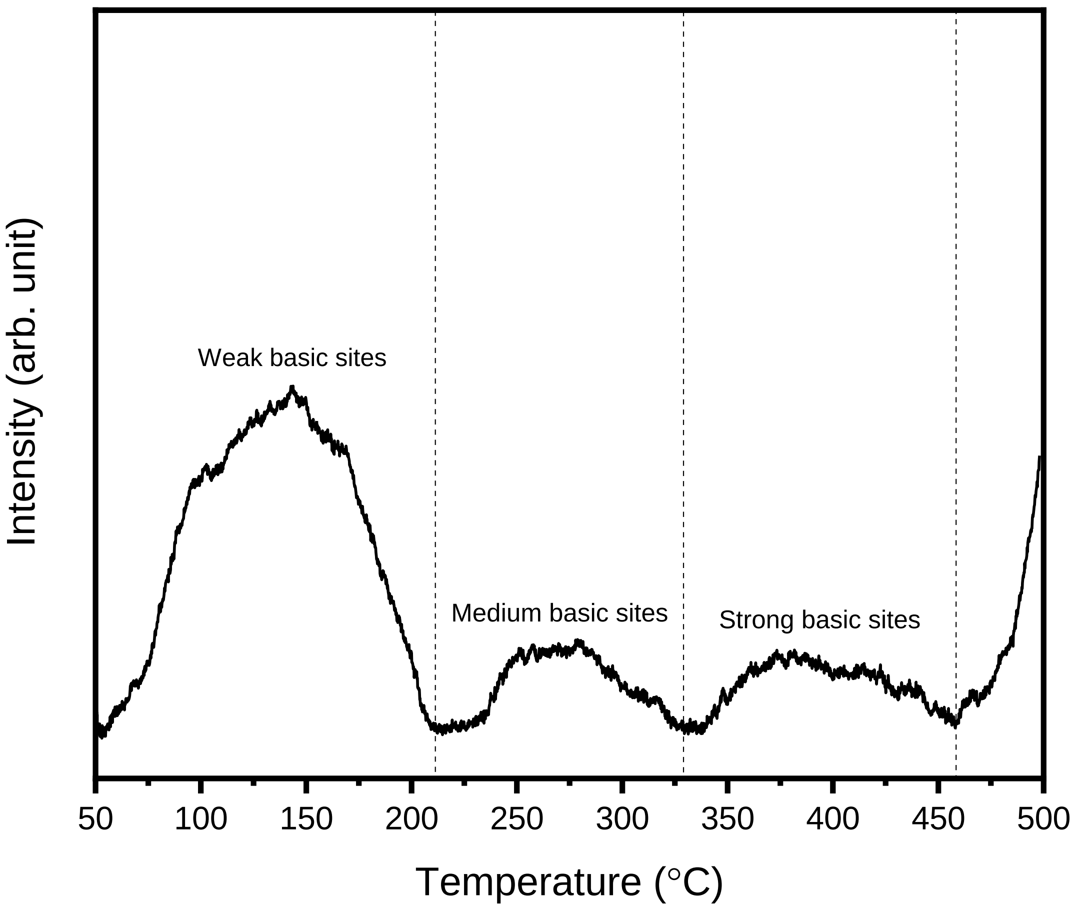

Fig. S5: CO_2-TPD analysis of T-MIL-88B-500

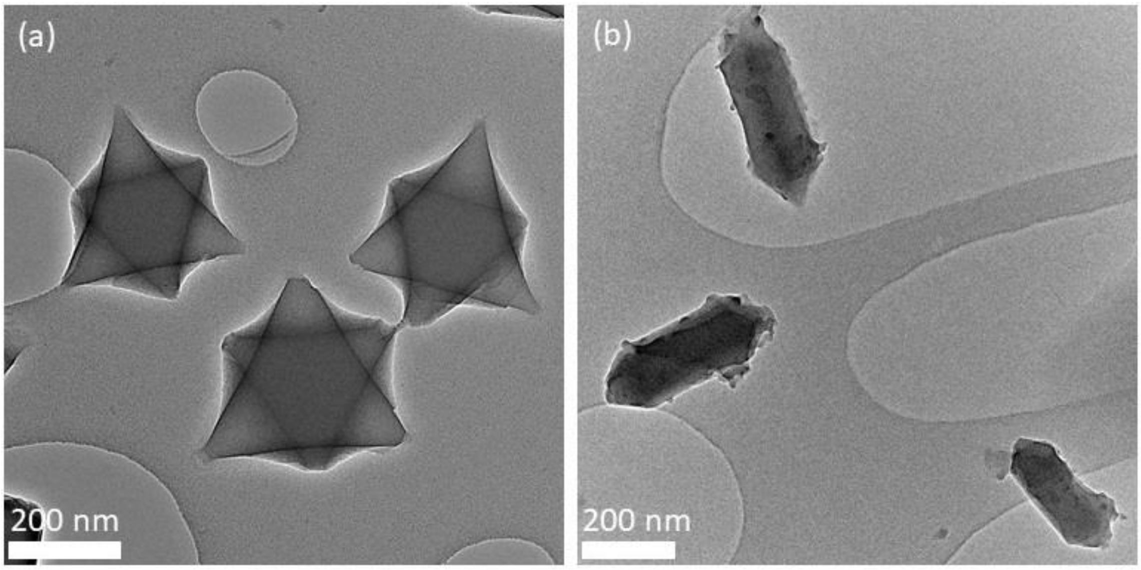

Fig. S6. TEM micrographs of synthesized (a) MIL-101 and (b) MIL-88B catalyst

Table S1: Elemental composition of thermally transformed MIL-88B (T-MIL-88B-500)

Elements	Weight percentages (%)
Fe*	49.3
Na*	19.5
C**	13.7
H**	0.6
N**	0.0
S**	0.0
O***	16.9

* measured by XRF analysis

** measured by CHNS analysis

*** calculated on the basis of difference

BET of studied catalysts

Figure S7.a-c demonstrates the nitrogen linear isotherms of different iron based CBEA, MIL-101 and MIL-88B catalysts, respectively. The nitrogen sorption of CBEA and Fe/CBEA catalyst (Figure S7.a) represent the combination of type I and IV isotherm which interpreted that the catalyst mostly consisted with micropores [1, 2]. The two steps nitrogen isotherm has been indicated that the presence of both micropores and mesopores in the catalyst. The micropores filling is mainly occurred in the ranges of 0<p/p°<0.05 and the mesoporous filling which leads to the appearance of hysteresis loop (H3 type) has been noticed in the ranges of 0.4<p/p°<0.9. The surface area of CBEA and reduced Fe/CBEA are 673.9 and 390.1 m^2/g, respectively. The surface area of reduced Fe/CBEA is lower than the CBEA support itself which is most likely the blockage of the pore with iron. The BJH pore size distribution of CBEA support (Figure S7.d) has been revealed that the presence of mesopores in between the ranges of 1.9-50 nm. The pore diameter and the mesopore volume of this support (CBEA) is 5.3 nm and 0.11 cm^3/g, respectively which has been changed minorly after the impregnation of iron as shown in Table S2. Whereas, the micropore volume of the CBEA support has changed significantly from 0.21 to 0.12 cm^3/g in reduced Fe/CBEA catalyst.

The N_2 adsorption-desorption isotherm of MIL-101 displayed typical type I isotherm along with the secondary uptakes at p/p° ~ 0.1 and p/p° ~ 0.2 which is good agreement with the previously reported results by Fe´rey et al. [3]. Fe/MIL-101 also exhibits the similar type of isotherm as compared to MIL-101. After impregnation of Fe in MIL-101, the surface area of the catalyst decreased from 2473.7 cm^2/g to 1831.3 cm^2/g which is maybe due to the blocking of the pores with metal that is enforced by the BJH results of these two catalysts. The micropores and mesopores volume of Fe/MIL-101 are 0.09 and 0.51 cm^3/g respectively which are comparatively lower than the micropore (0.35 cm^3/g) and mesopore (0.60 cm^3/g) volume of MIL-101

due to pore blockage. The total pore volume of Fe/MIL-101 is also lower than MIL-101 (Table S2). The degraded Fe/MIL-101 (T-Fe/MIL-101) catalyst shows (Figure S7.b) type II isotherm with H3 hysteresis loop which has a good resemblance with the results obtained for MIL-101(Cr)/RGO/$ZnFe_2O_4$ nanocomposite [4]. The surface area of T-Fe/MIL-101 is very lower (73.5 m^2/g) as compared to MIL-101 and Fe/MIL-101 which has a good resemblance with the findings obtained by Farisabadi et al. [5]. The poor surface area of this aforementioned catalyst is most likely due to the collapse of organic framework which was previously articulated by Li et al. for MIL-101-Cr MOF that was thermally treated at 550 °C [6]. In the low-pressure region, the absence of adsorption suggested that all micropores in MIL-101 are collapsed and the existence of hysteresis loop in the high-pressure region indicated the presence of mesopores [6].

Figure S7.c represented N_2 adsorption-desorption isotherm of MIL-88B and T-MIL-88B-500. MIL-88B exhibits the type I isotherm that suggesting the microporous MOF with high surface area (410.6 m^2/g) which is almost similar with the results reported by Vu et al. [7]. The pore size distribution of MIL-88B is displayed in Figure S7.f and the average pore diameter is 6.1 nm with total pore volume (both micropore and mesopore) of 0.32 cm^3/g. The N_2 adsorption-desorption isotherm of T-MIL-88B-500 is type II with H2 (b)-type hysteresis loop which is identical with the results obtained for MIL-88B that was thermally degraded at 500 °C under N_2 atmosphere [8]. Due to the presence of mesopores, the high-pressure region of the isotherm consisted with hysteresis loop. Table S2 showed that the surface area of T-MIL-88B-500 catalyst is lower (160.6 m^2/g) than MIL-88B which is most likely due to collapse of organic matrix [9].

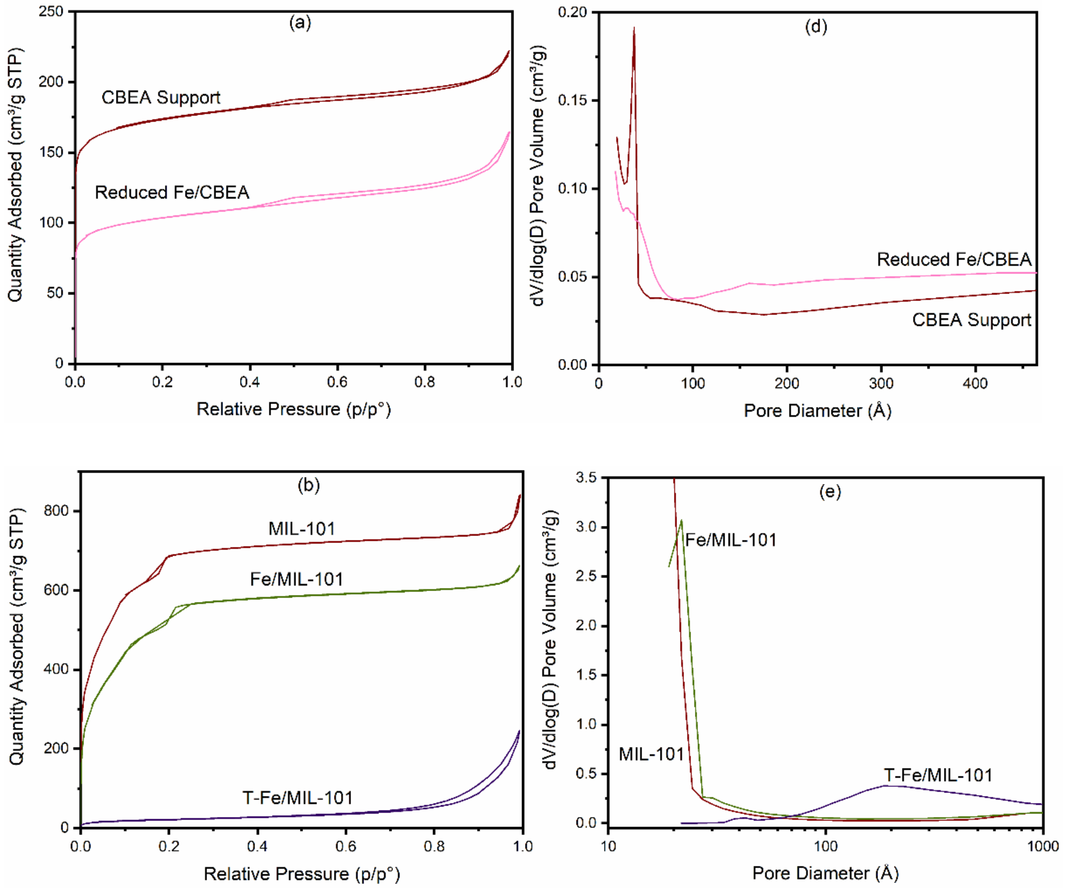

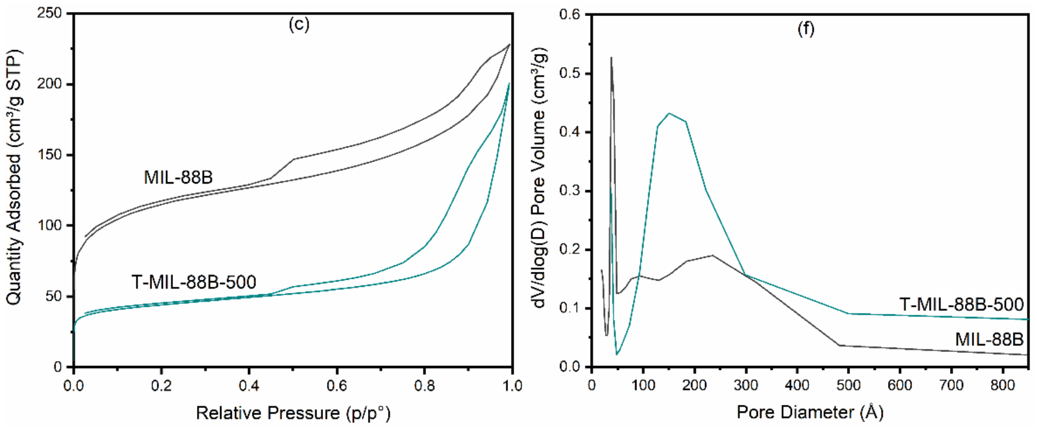

Fig. S7. Nitrogen sorption isotherms of (a) CBEA, (b) MIL-101, and (c) MIL-88B based Fe catalysts, respectively; and BJH desorption pore distribution of (d) CBEA, (e) MIL-101, and (f) MIL-88B based Fe catalysts, respectively.

Table S2: Physical and textural characteristics of studied supports and catalysts

Nanomaterials	†S_{BET}	††D_{Pore}	⊦V_{micro}	⊦⊦V_{meso}	⊦⊦⊦V_{total}
	(m^2/g)	(nm)	(cm^3/g)	(cm^3/g)	(cm^3/g)
CBEA	673.9	5.3	0.21	0.11	0.32
Reduced 10%Fe/CBEA	390.1	6.4	0.12	0.13	0.25
MIL-101	2473.7	3.0	0.35	0.60	0.95
10%Fe/MIL-101	1831.3	2.8	0.09	0.51	0.60
T-10%Fe/MIL-101	73.5	20.2	0.00	0.38	0.38
MIL-88B	410.6	6.1	0.09	0.23	0.32
T-MIL-88B-500	160.6	14.2	0.04	0.27	0.31

† surface area via Brunauer-Emmett-Teller (BET) method

†† Mean desorption diameter of pore via Barrett-Joyner-Halenda (BJH) method

⊦ Cumulative desorption pore volume via Barrett-Joyner-Halenda (BJH) method

⊦⊦ Micropore volume via t-plot

⊦⊦⊦ Total volume of pores

GC chromatograms for gaseous products

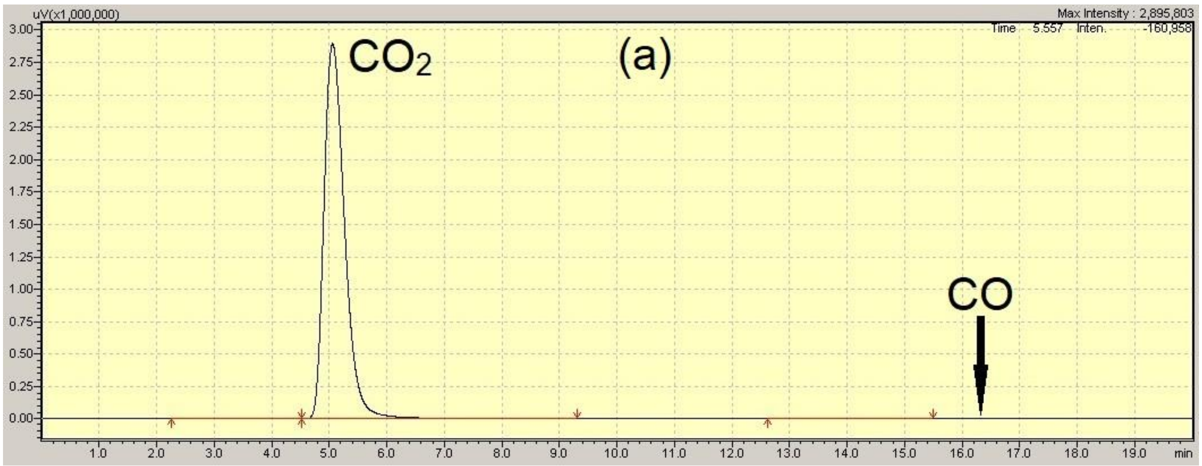

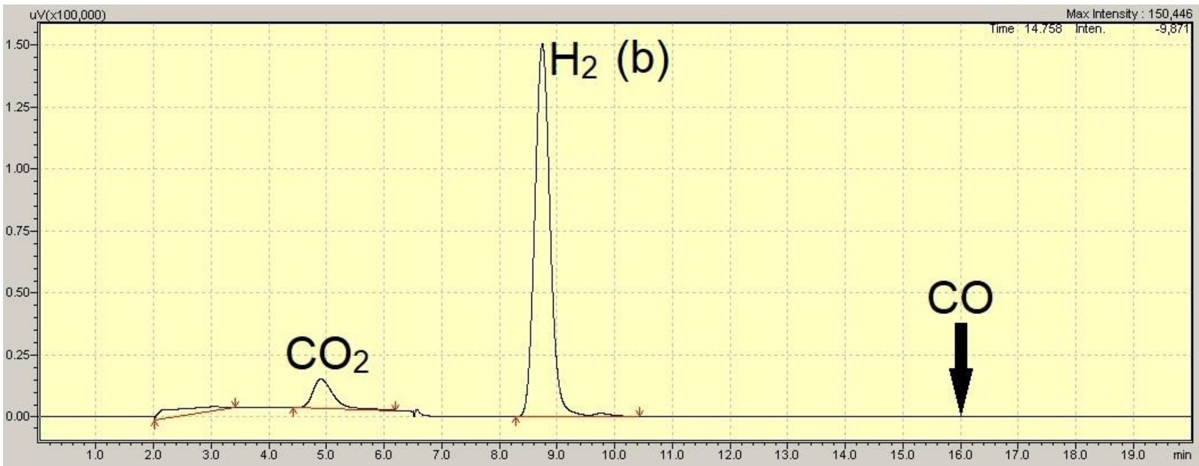

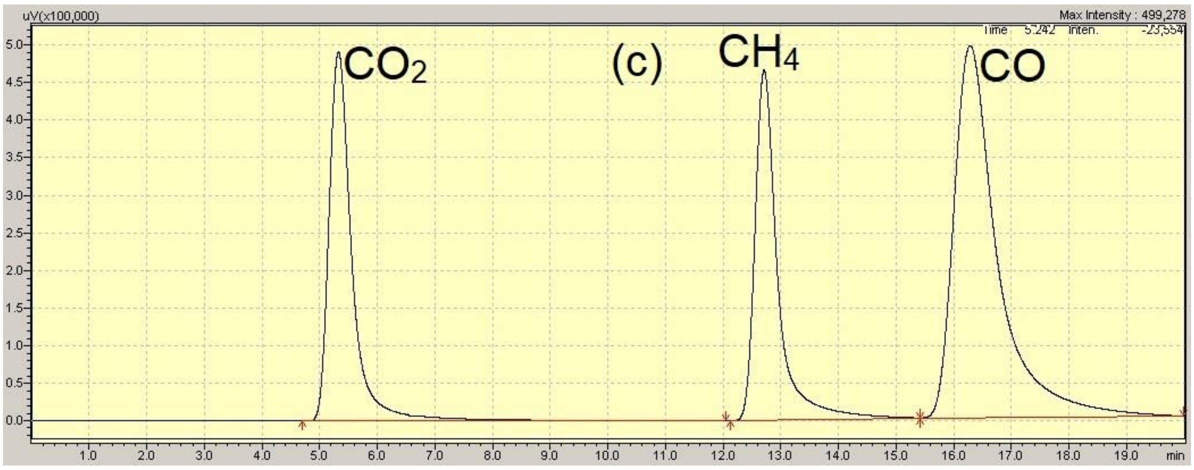

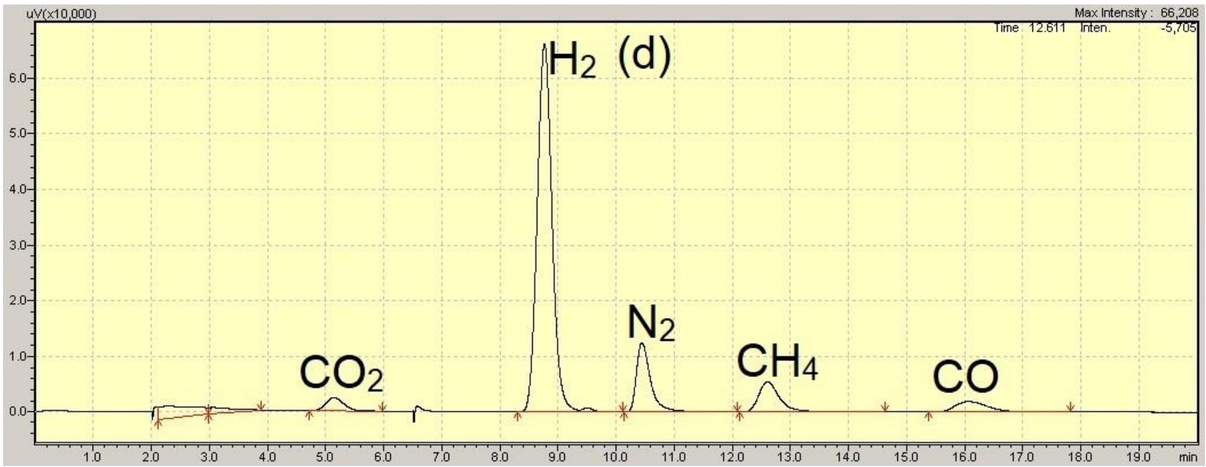

Fig. S8. GC chromatograms of (a) FID and (b) TCD detectors for gas sample of aqueous phase CO_2 hydrogenation using T-MIL-88B-500 in the presence of CH_3OH and LiI. Reaction conditions: T= 150 ˚C, H_2/CO_2= 1, t_R= 48h, P_{total}= 70 bar at room temperature and stirring speed= 200 RPM. GC chromatograms of (c) FID and (d) TCD detectors for gas sample of standard calibration gas mixture. Composition of standard calibration gas mixture: 20.20% CO, 20.43% H_2, 10.08% CH_4, 10.09% CO_2 and 39.2% N_2.

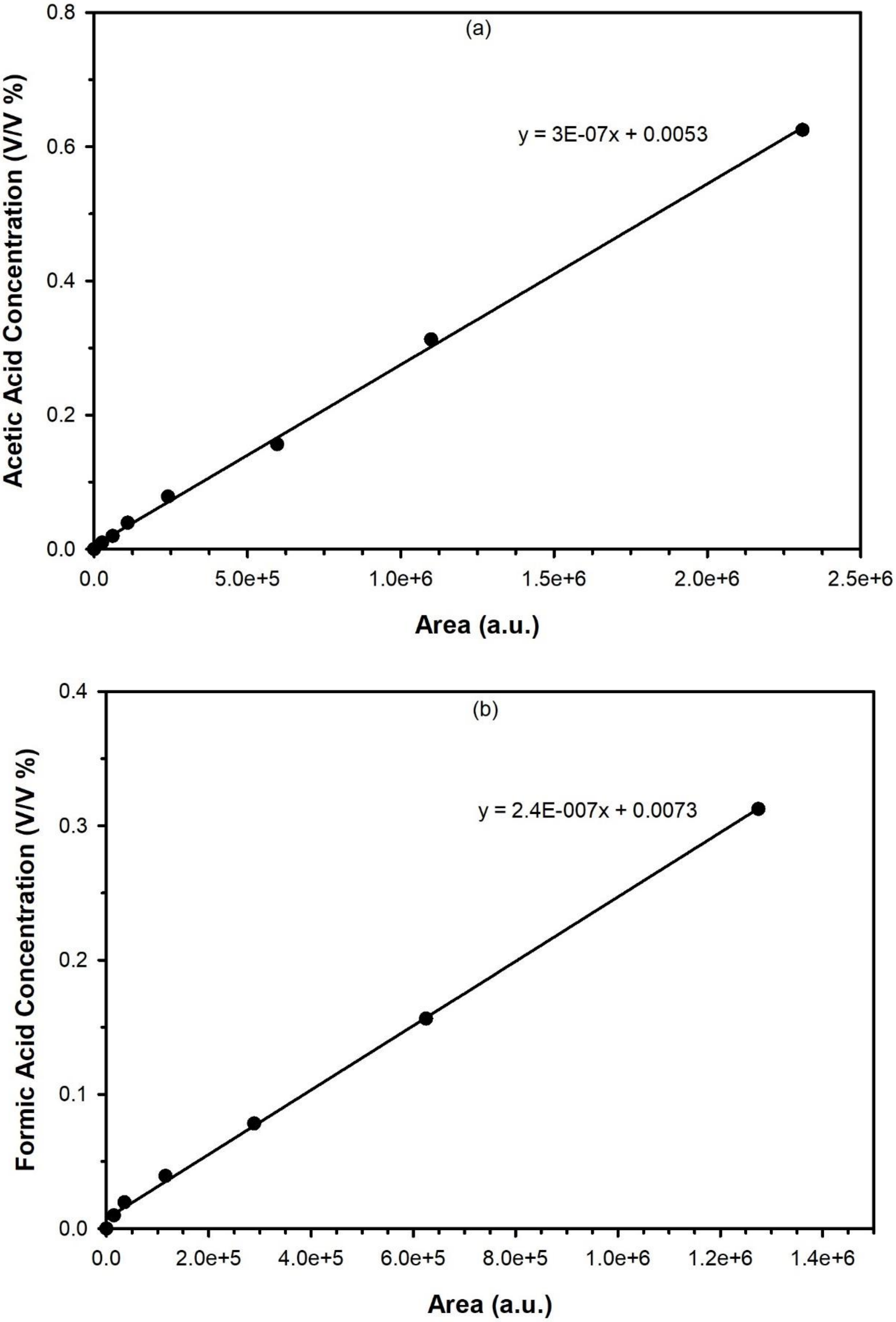

Fig. S9. Standard calibration curves of (a) acetic acid and (b) formic acid in Milli-Q water

References

1. Ahmad W, Chan FL, Chaffee AL, Wang H, Hoadley A, Tanksale A. Dimethoxymethane production via catalytic hydrogenation of carbon monoxide in methanol media. *ACS Sustainable Chemistry & Engineering* **8**, 2081-2092 (2020).

2. Ahmad W, Chan FL, Hoadley A, Wang H, Tanksale A. Synthesis of oxymethylene dimethyl ethers (OMEn) via methanol mediated COx hydrogenation over Ru/BEA catalysts. *Applied Catalysis B: Environmental* **269**, 118765 (2020).

3. Férey G*, et al.* A chromium terephthalate-based solid with unusually large pore volumes and surface area. *Science* **309**, 2040-2042 (2005).

4. Nirumand L, Farhadi S, Zabardasti A, Khataee A. Synthesis and sonocatalytic performance of a ternary magnetic MIL-101 (Cr)/RGO/ZnFe2O4 nanocomposite for degradation of dye pollutants. *Ultrasonics sonochemistry* **42**, 647-658 (2018).

5. Farisabadi A, Moradi M, Hajati S, Kiani MA, Espinos JP. Controlled thermolysis of MIL-101 (Fe, Cr) for synthesis of FexOy/porous carbon as negative electrode and Cr2O3/porous carbon as positive electrode of supercapacitor. *Applied Surface Science* **469**, 192-203 (2019).

6. Li X, Zhang L, Sun Y. Titanium-Modified MIL-101 (Cr) Derived Titanium-Chromium-Oxide as Highly Efficient Oxidative Desulfurization Catalyst. *Catalysts* **10**, 1091 (2020).

7. Vu TA*, et al.* Highly photocatalytic activity of novel Fe-MIL-88B/GO nanocomposite in the degradation of reactive dye from aqueous solution. *Materials Research Express* **4**, 035038 (2017).

8. Liu J*, et al.* Fe-MOF-derived highly active catalysts for carbon dioxide hydrogenation to valuable hydrocarbons. *Journal of CO2 Utilization* **21**, 100-107 (2017).

9. Yao X, Bai C, Chen J, Li Y. Efficient and selective green oxidation of alcohols by MOF-derived magnetic nanoparticles as a recoverable catalyst. *RSC advances* **6**, 26921-26928 (2016).
