# Supplementary material for: Aqueous phase conversion of CO2 into acetic acid over thermally transformed MIL-88B catalyst
Source: Nat Commun. 2023 May 17;14:2821. doi: 10.1038/s41467-023-38506-5 (PMC10192334; doi:10.1038/s41467-023-38506-5)
Supplement: Supplementary file 3 — Description of Additional Supplementary Files [file 41467_2023_38506_MOESM3_ESM.docx]

Supplementary Data 1: ReaxFF forcefield parameters for Fe/C/H/O
