# Supplementary material for: Aqueous phase conversion of CO2 into acetic acid over thermally transformed MIL-88B catalyst
Source: Nat Commun. 2023 May 17;14:2821. doi: 10.1038/s41467-023-38506-5 (PMC10192334; doi:10.1038/s41467-023-38506-5)
Supplement: Supplementary file 4 — Supplementary Data 1 [file 41467_2023_38506_MOESM4_ESM.pdf]

Reactive MD-force field: Fe/C/O/H

```
39       ! Number of general parameters
  50.0000 !Overcoordination parameter
   9.5469 !Overcoordination parameter
   1.6725 !Valency angle conjugation parameter
   1.7224 !Triple bond stabilisation parameter
   6.8702 !Triple bond stabilisation parameter
  60.4850 !C2-correction
   1.0588 !Undercoordination parameter
   4.6000 !Triple bond stabilisation parameter
  12.1176 !Undercoordination parameter
  13.3056 !Undercoordination parameter
 -55.1978 !Triple bond stabilization energy
   0.0000 !Lower Taper-radius
  10.0000 !Upper Taper-radius
   2.8793 !Not used
  33.8667 !Valency undercoordination
   6.0891 !Valency angle/lone pair parameter
   1.0563 !Valency angle
   2.0384 !Valency angle parameter
   6.1431 !Not used
   6.9290 !Double bond/angle parameter
   0.3989 !Double bond/angle parameter: overcoord
   3.9954 !Double bond/angle parameter: overcoord
  -2.4837 !Not used
   5.7796 !Torsion/BO parameter
  10.0000 !Torsion overcoordination
   1.9487 !Torsion overcoordination
  -1.2327 !Conjugation 0 (not used)
   2.1645 !Conjugation
   1.5591 !vdWaals shielding
   0.1000 !Cutoff for bond order (*100)
   1.7602 !Valency angle conjugation parameter
   0.6991 !Overcoordination parameter
  50.0000 !Overcoordination parameter
   1.8512 !Valency/lone pair parameter
   0.5000 !Not used
  20.0000 !Not used
   5.0000 !Molecular energy (not used)
   0.0000 !Molecular energy (not used)
   0.7903 !Valency angle conjugation parameter
  8    ! Nr of atoms; cov.r; valency;a.m;Rvdw;Evdw;gammaEEM;cov.r2;#
            alfa;gammavdW;valency;Eunder;Eover;chiEEM;etaEEM;n.u.
            cov r3;Elp;Heat inc.;n.u.;n.u.;n.u.;n.u.
            ov/un;val1;n.u.;val3,vval4
 C    1.3817   4.0000  12.0000   1.8903   0.1838   0.9000   1.1341   4.0000
      9.7559   2.1346   4.0000  34.9350  79.5548   5.9666   7.0000   0.0000
      1.2114   0.0000 202.6057   8.9539  34.9289  13.5366   0.8563   0.0000
     -2.8983   2.5000   1.0564   4.0000   2.9663   0.0000   0.0000   0.0000
 H    0.8930   1.0000   1.0080   1.3550   0.0930   0.8203  -0.1000   1.0000
      8.2230  33.2894   1.0000   0.0000 121.1250   3.7248   9.6093   1.0000
     -0.1000   0.0000  61.6606   3.0408   2.4197   0.0003   1.0698   0.0000
    -19.4571   4.2733   1.0338   1.0000   2.8793   0.0000   0.0000   0.0000
 O    1.2450   2.0000  15.9990   2.3890   0.1000   1.0898   1.0548   6.0000
      9.7300  13.8449   4.0000  37.5000 116.0768   8.5000   8.3122   2.0000
      0.9049   0.4056  59.0626   3.5027   0.7640   0.0021   0.9745   0.0000
     -3.5500   2.9000   1.0493   4.0000   2.9225   0.0000   0.0000   0.0000
```

```
Fe   1.9306   3.0000  55.8450   2.1229   0.1163   0.4744  -1.6836   3.0000
    10.4193   7.0680   3.0000   0.0000  18.3725   1.7785   8.6281   0.0000
    -1.2000   0.0000  66.4838  25.3430  10.1260   0.7590   0.8563   0.0000
   -16.0573   2.6997   1.0338   6.0000   2.5791   0.0000   0.0000   0.0000
Cl   1.7140   1.0000  35.4500   1.9139   0.2000   0.3837  -1.0000   7.0000
    11.5345  10.1330   1.0000   0.0000   0.0000   9.9614   6.5316   0.0000
    -1.0000   3.5750 143.1770   6.2293   5.2294   0.1542   0.8563   0.0000
   -10.2080   2.9867   1.0338   6.2998   2.5791   0.0000   0.0000   0.0000
Si   2.1932   4.0000  28.0600   1.8951   0.1737   0.8112   1.2962   4.0000
    11.3429   5.2054   4.0000  21.7115 139.9309   4.0081   5.7104   0.0000
    -1.0000   0.0000 128.2031   9.0751  23.8188   0.8381   0.8563   0.0000
    -4.1684   2.0754   1.0338   4.0000   2.5791   1.4000   0.2000  13.0000
Al   2.1967   3.0000  26.9820   2.3738   0.2328   0.4265  -1.6836   3.0000
     9.4002   3.9009   3.0000   0.0076  16.5151   1.7429   6.8319   0.0000
    -1.0000   0.0000  78.4675  20.0000   0.2500   0.0000   0.8563   0.0000
   -22.7101   1.7045   1.0338   8.0000   2.5791   1.4000   0.2000  13.0000
X   -0.1000   2.0000   1.0080   2.0000   0.0000   0.4744  -0.1000   6.0000
    10.0000   2.5000   4.0000   0.0000   0.0000   4.0000  15.0000   0.0000
    -0.1000   0.0000 127.6226   8.7410  13.3640   0.6690   0.9745   0.0000
   -11.0000   2.7466   1.0338   6.2998   2.8793   0.0000   0.0000   0.0000
25      ! Nr of bonds; Edis1;LPpen;n.u.;pbe1;pbo5;13corr;pbo6
                         pbe2;pbo3;pbo4;Etrip;pbo1;pbo2;ovcorr
  1  1 158.2004  99.1897  78.0000  -0.7738  -0.4550   1.0000  37.6117   0.4147
         0.4590  -0.1000   9.1628   1.0000  -0.0777   6.7268   1.0000   0.0000
  1  2 169.4760   0.0000   0.0000  -0.6083   0.0000   1.0000   6.0000   0.7652
         5.2290   1.0000   0.0000   1.0000  -0.0500   6.9136   0.0000   0.0000
  2  2 153.3934   0.0000   0.0000  -0.4600   0.0000   1.0000   6.0000   0.7300
         6.2500   1.0000   0.0000   1.0000  -0.0790   6.0552   0.0000   0.0000
  1  3 164.4303  82.6772  60.8077  -0.3739  -0.2351   1.0000  10.5036   1.0000
         0.4475  -0.2288   7.0250   1.0000  -0.1363   4.8734   0.0000   0.0000
  3  3 142.2858 145.0000  50.8293   0.2506  -0.1000   1.0000  29.7503   0.6051
         0.3451  -0.1055   9.0000   1.0000  -0.1225   5.5000   1.0000   0.0000
  2  3 160.0000   0.0000   0.0000  -0.5725   0.0000   1.0000   6.0000   0.5626
         1.1150   1.0000   0.0000   0.0000  -0.0920   4.2790   0.0000   0.0000
  1  4 113.1509   0.0000   0.0000   0.6400  -0.3000   1.0000  36.0000   0.0100
         1.0000  -0.3500  15.0000   1.0000  -0.1450   4.1504   1.0000   0.0000
  2  4  78.2669   0.0000   0.0000   0.4668   0.0000   1.0000   6.0000   0.1766
         0.5673   1.0000   0.0000   1.0000  -0.1543   5.4965   0.0000   0.0000
  3  4  77.2864   0.0000   0.0000   0.3288  -0.3000   1.0000  36.0000   0.1023
         1.0000  -0.3500  15.0000   1.0000  -0.1109   7.6514   1.0000   0.0000
  4  4  44.2147   0.0000   0.0000   0.2236  -0.2000   0.0000  16.0000   0.2849
         0.4922  -0.2000  15.0000   1.0000  -0.0552   6.7583   0.0000   0.0000
  2  5 109.1686   0.0000   0.0000  -0.1657  -0.2000   0.0000  16.0000   1.2500
         2.8463  -0.2000  15.0000   1.0000  -0.1111   5.2687   0.0000   0.0000
  3  5   0.0000   0.0000   0.0000   0.5000  -0.2000   0.0000  16.0000   0.5000
         1.0001  -0.2000  15.0000   1.0000  -0.1000  10.0000   0.0000   0.0000
  4  5   0.0000   0.0000   0.0000   0.2500  -0.2000   0.0000  16.0000   0.5000
         0.5000  -0.2000  15.0000   1.0000  -0.2000  10.0000   0.0000   0.0000
  5  5   0.2500   0.0000   0.0000   0.1803  -0.2000   0.0000  16.0000   0.3356
         0.9228  -0.2000  15.0000   1.0000  -0.1178   5.6715   0.0000   0.0000
  1  6   0.0000   0.0000   0.0000  -0.6528  -0.3000   0.0000  36.0000   0.5000
        10.0663  -0.3500  25.0000   1.0000  -0.1000  10.0000   0.0000   0.0000
  2  6 250.0000   0.0000   0.0000  -0.7128   0.0000   1.0000   6.0000   0.1186
        18.5790   1.0000   0.0000   1.0000  -0.0731   7.4983   0.0000   0.0000
  3  6 261.9074   5.9533   0.0000  -0.6223  -0.3000   1.0000  36.0000   0.7275
        10.1541  -0.2366  29.7817   1.0000  -0.1083   8.5924   6.0658   0.0000
  4  6   0.0000   0.0000   0.0000   1.0000   0.3000   0.0000  26.0000   1.0000
```

```
      0.5000   0.0000  12.0000   1.0000  -0.2000  10.0000   0.0000   0.0000
  6  6  70.9120  54.0531  30.0000   0.4931  -0.3000   1.0000  16.0000   0.0392
      0.2476  -0.8055   7.1248   1.0000  -0.1009   8.7229   0.0000   0.0000
  1  7   0.0000   0.0000   0.0000  -0.6528  -0.3000   0.0000  36.0000   0.5000
     10.0663  -0.3500  25.0000   1.0000  -0.1000  10.0000   0.0000   0.0000
  2  7   0.8579   0.0000   0.0000  -0.6528  -0.3000   0.0000  36.0000   0.1551
     10.0663  -0.3500  25.0000   1.0000  -0.0842   7.1758   0.0000   0.0000
  3  7 227.9327   0.0000   0.0000  -0.8375  -0.3000   0.0000  36.0000   0.1286
      0.3686  -0.3500  25.0000   1.0000  -0.1740   5.2057   0.0000   0.0000
  4  7   0.0000   0.0000   0.0000   1.0000   0.3000   0.0000  26.0000   1.0000
      0.5000   0.0000  12.0000   1.0000  -0.2000  10.0000   0.0000   0.0000
  6  7   0.0000   0.0000   0.0000   1.0000   0.3000   0.0000  26.0000   1.0000
      0.5000   0.0000  12.0000   1.0000  -0.2000  10.0000   0.0000   0.0000
  7  7  34.0777   0.0000   0.0000   0.4832  -0.3000   0.0000  16.0000   0.5154
      6.4631  -0.4197  14.3085   1.0000  -0.1463   6.1608   0.0000   0.0000
 17    ! Nr of off-diagonal terms; Ediss;Ro;gamma;rsigma;rpi;rpi2
  1  2   0.1239   1.4004   9.8467   1.1210  -1.0000  -1.0000
  2  3   0.0283   1.2885  10.9190   0.9215  -1.0000  -1.0000
  1  3   0.1345   1.8422   9.7725   1.2835   1.1576   1.0637
  1  4   0.3999   1.4558  11.0036   1.3918  -1.0000  -1.0000
  2  4   0.0200   1.9451  10.8595   1.4157  -1.0000  -1.0000
  3  4   0.0502   1.3407  10.1767   1.7735  -1.0000  -1.0000
  2  5   0.0568   1.6740   9.6297   1.2200  -1.0000  -1.0000
  3  5   0.1927   2.2551  11.2308  -1.0000  -1.0000  -1.0000
  4  5   0.1500   2.1500  11.0000  -1.0000  -1.0000  -1.0000
  1  6   0.2000   1.9000  12.0000  -1.0000  -1.0000  -1.0000
  2  6   0.1043   1.5000  11.8720   1.3005  -1.0000  -1.0000
  3  6   0.2000   1.9048  10.8374   1.7163   1.2444  -1.0000
  4  6   0.1000   2.0000  11.0000  -1.0000  -1.0000  -1.0000
  1  7   0.2000   1.9000  12.0000  -1.0000  -1.0000  -1.0000
  2  7   0.0564   1.4937  12.0744   1.7276  -1.0000  -1.0000
  3  7   0.1745   1.8928  11.2476   1.5382  -1.0000  -1.0000
  6  7   0.0295   1.5025  11.8687  -1.0000  -1.0000  -1.0000
 66    ! Nr of angles;at1;at2;at3;Thetao,o;ka;kb;pv1;pv2
  1  1  1  59.0573  30.7029   0.7606   0.0000   0.7180   6.2933   1.1244
  1  1  2  65.7758  14.5234   6.2481   0.0000   0.5665   0.0000   1.6255
  2  1  2  70.2607  25.2202   3.7312   0.0000   0.0050   0.0000   2.7500
  1  2  2   0.0000   0.0000   6.0000   0.0000   0.0000   0.0000   1.0400
  1  2  1   0.0000   3.4110   7.7350   0.0000   0.0000   0.0000   1.0400
  2  2  2   0.0000  27.9213   5.8635   0.0000   0.0000   0.0000   1.0400
  1  1  3  53.9517   7.8968   2.6122   0.0000   3.0000  58.6562   1.0338
  3  1  3  76.9627  44.2852   2.4177 -25.3063   1.6334 -50.0000   2.7392
  2  1  3  65.0000  16.3141   5.2730   0.0000   0.4448   0.0000   1.4077
  1  3  1  72.6199  42.5510   0.7205   0.0000   2.9294   0.0000   1.3096
  1  3  3  81.9029  32.2258   1.7397   0.0000   0.9888  68.1072   1.7777
  3  3  3  80.7324  30.4554   0.9953   0.0000   3.0000  50.0000   1.0783
  1  3  2  70.1101  13.1217   4.4734   0.0000   0.8433   0.0000   3.0000
  2  3  3  75.6935  50.0000   2.0000   0.0000   1.0000   0.0000   1.1680
  2  3  2  85.8000   9.8453   2.2720   0.0000   2.8635   0.0000   1.5800
  1  2  3   0.0000  25.0000   3.0000   0.0000   1.0000   0.0000   1.0400
  3  2  3   0.0000  15.0000   2.8900   0.0000   0.0000   0.0000   2.8774
  2  2  3   0.0000   8.5744   3.0000   0.0000   0.0000   0.0000   1.0421
  1  4  1   7.0546  39.3227   1.0694   0.0000   0.7210   0.0000   3.1133
  1  1  4  29.2291  39.0677   1.2584   0.0000   0.0100   0.0000   1.6263
  1  4  4  47.3783   1.0170   7.8341   0.0000   2.8546   0.0000   1.0000
  4  1  4  33.2812  34.6443   3.0111   0.0000   0.1701   0.0000   1.0510
  2  1  4   0.0100  25.4983   1.5660   0.0000   0.0839   0.0000   1.2521
```

```
 2  4  2  20.3683   0.0100   2.2825   0.0000   0.7660   0.0000   1.3788
 2  2  4   0.0000   0.0100   1.0568   0.0000   1.8595   0.0000   3.6142
 4  2  4   0.0000  10.4428   7.9607   0.0000   2.3717   0.0000   1.1970
 2  4  4  48.4128   4.0632   0.6773   0.0000   2.2274   0.0000   1.8605
 2  4  4 180.0000  -5.3883  21.7804   0.0000   0.5866   0.0000   1.0000
 1  3  4  38.1871  14.3372   1.1301   0.0000   1.0108   0.0000   1.1000
 3  1  4  59.8640  25.0000   2.5000   0.0000   2.0000   0.0000   1.1000
 3  4  3  47.8282   0.0100   1.9197   0.0000   0.1248   0.0000   2.5839
 4  3  4  43.8679  14.3345   8.0000   0.0000   0.0636   0.0000   1.6997
 2  3  4  26.0012  49.6772   0.0500   0.0000   1.1589   0.0000   1.0000
 3  3  4  73.6721  32.6330   1.7223   0.0000   1.0221   0.0000   1.4351
 3  4  4  68.7142   3.0974   8.0000   0.0000   0.0907   0.0000   1.0000
 3  2  4   0.0000   0.0100   3.2567   0.0000   2.0582   0.0000   1.3513
 2  4  3  38.5594  11.2599   0.1898   0.0000   0.1904   0.0000   1.4041
 3  2  5   0.0000   0.0100   0.5211   0.0000   0.0000   0.0000   1.3859
 1  4  2  44.2808   0.3810   0.0100   0.0000   0.2603   0.0000   1.9721
 1  2  4   0.8171   0.0100   2.8321   0.0000   2.4786   0.0000   2.3245
 6  6  6  78.5339  36.4328   1.0067   0.0000   0.1694   0.0000   1.6608
 2  6  6  77.2616   5.0190   7.8944   0.0000   4.0000   0.0000   1.0400
 2  6  2  75.7983  14.4132   2.8640   0.0000   4.0000   0.0000   1.0400
 3  6  6  90.6812  31.1846   4.4543   0.0000   0.5073   0.0000   2.1809
 2  6  3  73.6998  40.0000   1.8782   0.0000   4.0000   0.0000   1.1290
 3  6  3  80.1361  36.2368   0.9504   0.0000   0.2624   0.0000   2.0787
 6  3  6  80.4450   6.0739   1.7731   0.0000   3.2548   0.0000   1.0422
 2  3  6  86.7611   7.1742   1.4013   0.0000   1.4999   0.0000   1.0400
 3  3  6 103.4529  26.9589   1.3470   0.0000   1.7728   0.0000   1.3091
 2  2  6   0.0000  47.1300   6.0000   0.0000   1.6371   0.0000   1.0400
 6  2  6   0.0000  27.4206   6.0000   0.0000   1.6371   0.0000   1.0400
 3  2  6   0.0000   5.0000   1.0000   0.0000   1.0000   0.0000   1.2500
 3  2  7   0.0000   4.2750   1.0250   0.0000   1.3750   0.0000   1.4750
 2  2  7   0.0000   3.0000   1.0000   0.0000   1.0000   0.0000   1.2500
 7  2  7   0.0000  20.2391   0.1328   0.0000   2.9860   0.0000   1.0870
 2  3  7  90.0000  19.7491   1.8227   0.0000   1.0000   0.0000   2.5337
 3  3  7  34.4326  25.9544   5.1239   0.0000   2.7500   0.0000   1.7141
 7  3  7  23.7270  19.5973   4.0000   0.0000   0.6619   0.0000   1.9380
 2  7  2  67.4229   4.5148   5.9702   0.0000   3.0000   0.0000   2.6879
 2  7  3  41.8108  17.3800   2.6618   0.0000   0.7372   0.0000   1.0100
 3  7  3  54.0864   9.7594   1.9476   0.0000   3.0000   0.0000   1.4400
 2  7  7 180.0000 -26.7860   7.3549   0.0000   1.0000   0.0000   1.0252
 2  7  7  78.2279  37.6504   0.4809   0.0000   1.0000   0.0000   2.9475
 6  3  7  15.7093   0.0100   2.7033   0.0000   1.0000   0.0000   1.0000
 3  6  7  88.2703   0.3954   0.2500   0.0000   0.5000   0.0000   2.1060
 3  7  6  83.8306   0.3712   0.2500   0.0000   0.5000   0.0000   2.1153
33    ! Nr of torsions;at1;at2;at3;at4;;V1;V2;V3;V2(BO);vconj;n.u;n
 1  1  1  1  -0.2500  34.7453   0.0288  -6.3507  -1.6000   0.0000   0.0000
 1  1  1  2  -0.2500  29.2131   0.2945  -4.9581  -2.1802   0.0000   0.0000
 2  1  1  2  -0.2500  31.2081   0.4539  -4.8923  -2.2677   0.0000   0.0000
 1  1  1  3   1.2799  20.7787  -0.5249  -2.5000  -1.0000   0.0000   0.0000
 2  1  1  3   1.9159  19.8113   0.7914  -4.6995  -1.0000   0.0000   0.0000
 3  1  1  3  -1.4477  16.6853   0.6461  -4.9622  -1.0000   0.0000   0.0000
 1  1  3  1   0.4816  19.6316  -0.0057  -2.5000  -1.0000   0.0000   0.0000
 1  1  3  2   1.2044  80.0000  -0.3139  -6.1481  -1.0000   0.0000   0.0000
 2  1  3  1  -2.5000  31.0191   0.6165  -2.7733  -2.9807   0.0000   0.0000
 2  1  3  2  -2.4875  70.8145   0.7582  -4.2274  -3.0000   0.0000   0.0000
 1  1  3  3  -0.3566  10.0000   0.0816  -2.6110  -1.9631   0.0000   0.0000
 2  1  3  3  -1.4383  80.0000   1.0000  -3.6877  -2.8000   0.0000   0.0000
 3  1  3  1  -1.1390  78.0747  -0.0964  -4.5172  -3.0000   0.0000   0.0000
```

```
3  1  3  2  -2.5000  70.3345  -1.0000  -5.5315  -3.0000   0.0000   0.0000
3  1  3  3  -2.0234  80.0000   0.1684  -3.1568  -2.6174   0.0000   0.0000
1  3  3  1   1.1637 -17.3637   0.5459  -3.6005  -2.6938   0.0000   0.0000
1  3  3  2  -2.1289  12.8382   1.0000  -5.6657  -2.9759   0.0000   0.0000
2  3  3  2   2.5000 -22.9397   0.6991  -3.3961  -1.0000   0.0000   0.0000
1  3  3  3   2.5000 -25.0000   1.0000  -2.5000  -1.0000   0.0000   0.0000
2  3  3  3  -2.5000  -2.5103  -1.0000  -2.5000  -1.0000   0.0000   0.0000
3  3  3  3  -2.5000 -25.0000   1.0000  -2.5000  -1.0000   0.0000   0.0000
0  1  2  0   0.0000   0.0000   0.0000   0.0000   0.0000   0.0000   0.0000
0  2  2  0   0.0000   0.0000   0.0000   0.0000   0.0000   0.0000   0.0000
0  2  3  0   0.0000   0.1000   0.0200  -2.5415   0.0000   0.0000   0.0000
0  1  1  0   0.0000  50.0000   0.3000  -4.0000   0.0000   0.0000   0.0000
0  3  3  0   0.5511  25.4150   1.1330  -5.1903  -1.0000   0.0000   0.0000
1  1  3  3  -0.0002  20.1851   0.1601  -9.0000  -2.0000   0.0000   0.0000
1  3  3  1   0.0002  80.0000  -1.5000  -4.4848  -2.0000   0.0000   0.0000
3  1  3  3  -0.1583  20.0000   1.5000  -9.0000  -2.0000   0.0000   0.0000
2  6  6  2   0.0000   0.0000   0.0640  -2.4426   0.0000   0.0000   0.0000
2  6  6  6   0.0000   0.0000   0.1587  -2.4426   0.0000   0.0000   0.0000
0  2  6  0   0.0000   0.0000   0.1200  -2.4847   0.0000   0.0000   0.0000
2  1  4  4   0.0000   0.0000   0.0000  -5.0000   0.0000   0.0000   0.0000
1    ! Nr of hydrogen bonds;at1;at2;at3;Rhb;Dehb;vhb1
3  2  3   2.1200  -3.5800   1.4500  19.5000
```
